# Molecular mechanism of CD44 homodimerization modulated by palmitoylation and membrane environments

**DOI:** 10.1016/j.bpj.2022.06.021

**Published:** 2022-06-22

**Authors:** Ziyi Ma, Sai Shi, Meina Ren, Chunli Pang, Yong Zhan, Hailong An, Fude Sun

**Affiliations:** 1 Key Laboratory of Molecular Biophysics, Hebei Province, Institute of Biophysics, School of Health Science & Biomedical Engineering, Hebei University of Technology, Tianjin, China; 2 State Key Laboratory of Reliability and Intelligence of Electrical Equipment, Hebei University of Technology, Tianjin, China; 3 Key Laboratory of Electromagnetic Field and Electrical Apparatus Reliability of Hebei Province, Hebei University of Technology, Tianjin, China

## Abstract

The homodimerization of CD44 plays a key role in an intercellular-to-extracellular signal transduction and tumor progression. Acylated modification and specific membrane environments have been reported to mediate translocation and oligomerization of CD44; however, the underlying molecular mechanism remains elusive. In this study, extensive molecular dynamics simulations are performed to characterize the dimerization of palmitoylated CD44 variants in different bilayer environments. CD44 forms homodimer depending on the cysteines on the juxta-membrane domains, and the dimerization efficiency and packing configurations are defected by their palmitoylated modifications. In the phase-segregated (raft included) membrane, homodimerization of the palmitoylated CD44 is hardly observed, whereas PIP2 addition compensates to realize dimerization. However, the structure of CD44 homodimer formed in the phase-segregated bilayer turns susceptive and PIP2 addition allows for an extensive conformation of the cytoplasmic domain, a proposal prerequisite to access the cytoskeleton linker proteins. The results unravel a delicate competitive relationship between PIP2, lipid raft, and palmitoylation in mediating protein homodimerization, which helps to clarify the dynamic dimer conformations and involved cellular signaling of the CD44 likewise proteins.

## Significance

It has been confirmed that CD44s form homodimerization as a precondition for an intercellular-to-extracellular signal transduction correlative with tumor progression. The bidirectional translocation of CD44 in membrane modulated by palmitoylation has been recently reported, whereas the dimerization philosophy of the palmitoylated CD44 in heterogeneity membrane remains unclear. Herein, the extensive molecular dynamic simulations are employed and the divergent influence of two palmitoyl modifications on CD44 dimerization is probed. This study further highlights the exceptional role of PIP2 in prompting CD44 dimerization that is substantially suppressed by lipid raft. Taken together, the manuscript provides molecular insights into the palmitoyl-modulated dimerization and packing conformations of CD44 in different membrane environments, which acts as key clues for cyto-protein linkage and signal transduction.

## Introduction

**Figure 1:**
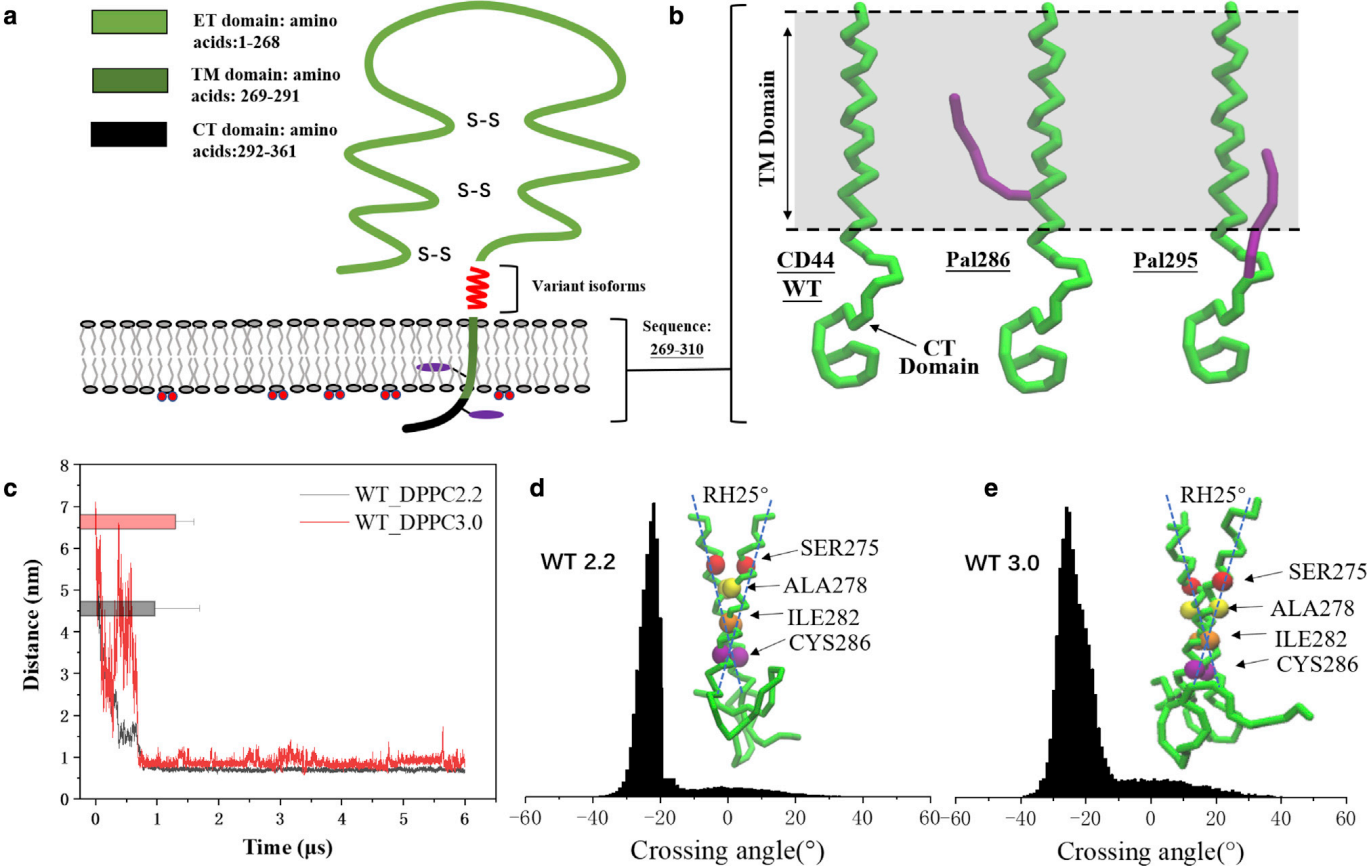
Presentations of palmitoylated CD44 models and dimerization of CD44-WT in DPPC bilayer. (*a*) CD44 is composed of the extracellular (ET) domain where the variant exon products (*red*) are inserted, the transmembrane (TM) domain and the cytoplasmic tail (CT). The palmitoyl moieties are shown by purple chains. (*b*) Presentation of CD44 CG models of the WT, Pal-286, and Pal-295. Only the backbone beads of CD44 are shown for clarity. The palmitoyl moieties are shown by purple chains, connecting to cysteines 286 and 295, respectively. (*c*) The distance evolvements between the TM domains averaged from six 6.0 *μ*s samples of the CD44-WT in 2.2 and 3.0 versions. Histograms and error bars were used to describe the average distance and standard deviation. The TM crossing angle distribution of CD44-WT in (*d*) the 2.2 version and (*e*) in the 3.0 version. The inserted panels represent the respective chiral dimer modes. Residues of SER, ALA, ILE, and CYS are presented as red, yellow, orange, and purple beads, respectively. The color methods remain consistent in the following parts without additive notation. More details can be seen in the Methods. To see this figure in color, go online.

Cluster of differentiation 44 (CD44) is a transmembrane glycoprotein and functions in various cellular processes, such as angiogenesis, bone metastasis, cell migration, and cancer invasiveness (1, 2, 3, 4). The extracellular domain (ETD) of CD44 undergoes binding of hyaluronic acid (HA) and triggers inhibition of cell proliferation, while an interrupted HA binding by ectodomain cleavage or inhibitors has been regarded as an antitumor strategy for high expression of CD44 in many tumor cells (5, 6, 7). As a single-pass transmembrane protein, a signal transduction termed the “inside-out” pathway is proposed: that proteolysis of the ETD depends on CD44 homodimerization and modifications of the cytoplasmic domain (CTD) (8). Substantial amounts of CD44 exist as homodimers on the cell surface. A recent study reveals that a suppressed CD44 dimerization reduces tumor cell aggregation in vitro (9). It is known the intercellular homo-interaction of the ETD happens to drive tumor clusters (10), while the intracellular CD44 dimerization is mainly dependent on the transmembrane domain (TMD) and CTD where the sequence is highly conserved (2) (Fig. 1 *a*). A previous study shows that CD44 dimerization requires a pair of double disulfide bridges established by two cysteines located at the TMD and the membrane proximate CTD, respectively (11). Existing evidence confirms the significant role of cysteine on the TMD in maintaining CD44 dimerization (8). Note that the intracellular homodimerization of CD44 performs as an assistor for HA binding of the ETD, in accordance with the dimer-stimulated tumor exasperation pathway.

It is noteworthy that the cysteine residues in the membrane proximate can be modified by palmitoylation, one of the most frequent acylation modifications observed for membrane proteins (12,13). Increasing evidence stresses the importance of palmitoylation in CD44 diffusion and protein package in the membrane (14, 15). The palmitoylation confers many transmembrane proteins of high affinity for the cholesterol-enriched membrane subdomain, termed lipid rafts (16,17). Palmitoylated CD44 harbored in lipid rafts is found to be unfavorable for the migration of breast tumor cells (7). Our recent study further reveals that raft attachment of palmitoylated CD44 leads to a reduced protein association with the active ERM N-terminal (FERM) domain (14). Note that the cysteine residues involved in dimerization are exactly the sites of palmitoylated modification; it is intriguing to make sense of whether palmitoylation and to what extent it influences the dimerization capability of CD44. Study of a single-span transmembrane linker for activation of T cells shows that palmitoylation on the juxta-membrane domain enhances its dimerization by partitioning in lipid rafts (16). Considering that palmitoylation of CD44 is unfavorable for cell migration, it is surmised that palmitoylation plays a different role in assembling with that of the linker for activation of T cells. Given that palmitoylation leads to a differential localization in a phase-segregated membrane for homologous proteins, such as N-Ras and K-Ras4b (18), the dimerization of CD44 affected by palmitoylation is necessary for probing in a more realistic membrane system.

Lipid-mediated assembling and signal transferring of TM proteins have been extensively reported. Studies have shown that a specific lipid, phosphatidylinositol 4,5-diphosphate (PIP2), has a significant affinity with the juxta-membrane domains of many TM proteins, which regulates protein aggregation, conformational switching, and signal transduction (19, 20, 21). Recent studies have revealed a positive role of PIP2 in assembly of CD44 and its association with FERM in solution (22). Despite the distribution of PIP2 relative to the lipid raft remains debated, the location preference of PIP2 in the nonraft subdomain has been supported by recent studies (20,23), basically due to the high degree of unsaturation in its *sn*-2 hydrocarbon tail. The exceptional role of PIP2 in controlling protein translocation and membrane binding orientation has also been profound for the focal adhesion kinase, a FERM-activated protein (24). Our recent work has proven that PIP2 enables the palmitoylated CD44 to release from the lipid raft (the liquid-ordered [*Lo*] subdomain was named the lipid raft because of its comparable lipid organization, while the liquid-disordered [*Ld*] subdomain was named the nonraft); therefore, it exerts an opposite effect to palmitoylation in mediating CD44 translation (14). Note that CD44 palmitoylated at different cysteines is observed to perform discrepant translocation capacity. This caused us to be curious as to how different palmitoylations affect its assembling ability, especially when modulated by the lipid nanodomain as well as PIP2 addition. To date, in-depth studies exploring the inherent correlation between CD44-like TM protein dimerization, acylation, and membrane translocation in molecular details has, however, not yet been reported.

To capture more insights into the dimerization and translocation of transmembrane proteins on a molecular level, Martini coarse-grained (CG) simulation has been extensively used in studying lipid-mediated protein assembling and protein modifications (25, 26, 27). Based on an average of four-to-one mapping in the integration of an all-atom model, the Martini CG force field not only ensures the accuracy in simulation but also effectively expands the system capacity and simulation duration. In the latest open-beta Martini 3.0 force field (28), the properties of CG beads and interacting matrix are substantially improved to realistically predict protein interactions, including transmembrane homodimerization, which makes the model increasingly adaptive and versatile (29). In addition to continuous updates, it has become more practical for capturing molecular interaction details by high-throughput dynamic simulation results (30,31). The Martini model has a wide range of applications, including membrane protein-protein association (32, 33, 34), protein-lipid interaction (35, 36, 37), and protein conformational change (24,38).

In this study, extensive dynamic simulations are employed to systematically explore the dimerization mechanism of CD44 under progressive environmental conditions. Firstly, CD44 alternatively palmitoylated at two cysteines in the pure dipalmitoyl-phosphatidylcholine (DPPC) bilayer model are subjected to simulations to analyze the impact of palmitoylation at different positions on dimerization. Secondly, PIP2 lipids are recruited in the DPPC bilayer to estimate the dimerization ability and conformational characteristics of CD44 series. Thirdly, the phase-segregated bilayer is introduced to explore the influence of the lipid raft on CD44 dimerization, as well as its translation behavior. Again, PIP2 is introduced to explore the possible changes of CD44 dimerization modulated by switching membrane translation. We believe that these molecular insights are conducive to a broader understanding of self-assembly and signal transduction of CD44-like TM proteins influenced by palmitoylations and membrane microenvironments.

## Methods

### System setup

The membrane model was constructed with the *insane* script. The model for CD44 (^269^WLIILASLLALALILAVCIAVNSRRRCGQKKKLVINSGNGAV^310^) used throughout the article consists of the TM domain (underlined) and a truncated CT domain. The CT domain containing 19 residues has been identified to interact with the actin linker protein (39). The initial structure of the protein was constructed with the software PYMOL (40) and then transferred into the CG structure with the *martinize* tool for version 2.2 and *martinize 2* tools for version 3.0. The secondary structure of the CD44-TM domain was defined as an α-helix, while the CT domain is a random structure, as revealed in a previous study. The palmitoyl moieties attached to the side chains of Cys-286 and Cys-295 were respectively constructed as shown in Fig. 1 *a*, and the modeling parameters of the palmitoylation remain identical to our previous study (25). Two CD44 monomers were inserted parallel to the membrane normal with the TM domain spanning through the bilayer, thereby exposing the CT domain in the intracellular domain. The initial distance between two peptides was set as 55 Å in the pure DPPC- and PIP2-containing membranes to avoid a noncovalent interaction between the peptides in the initial state, while the distance was elongated to 95 Å in the raft-formed membrane. In all cases, the membranes were solvated with water and counter ions.

### Simulation details

All simulations were performed in the software package of GROMACS-5.1.2 (41). All the snapshot presentations in this study were made using the VMD software package (42).

Martini 2.2 parameters: energy minimization was first conducted for the membrane-protein systems. The steepest descent method was used to minimize the system energy for 5000 steps. Before the preequilibration, the system was grouped into “proteins,” “lipids,” and “solvent” to ensure a smooth energy coupling process. The NVT equilibration was carried on for a duration of 10 ns using the Berendsen constant temperature coupling method (43), with a reference temperature of 323 K and a time constant of 1.0 ps. The NPT equilibration process uses Berendsen constant pressure coupling, using a semi-isotropic coupling type with a reference pressure of 1.0 bar, a compression constant of 4.5 × 10^−5^ bar^−1^, and a time constant of 5.0 ps for a time duration of 50 ns. In the equilibration processes, the protein backbone restraints, with a constant force of 1000 kJ · mol^−1^ · nm^−2^, were used to keep the protein from moving. For the raft-forming system, the temperature path used a reference temperature of 295 K and a time constant of 1.0 ps with v-rescale coupling methods. Meanwhile, the pressure coupling type chosen was the Parrinello-Rahman method to adapt the conditions in the formation of the phase-segregated bilayer. A shift cutoff method was applied for the nonbonded interactions such that the Coulomb and van der Waals interactions decreased to zero at 1.2 nm from 0.0 to 0.9 nm, respectively. After equilibration, the simulations ran with the periodic boundary conditions and a time step of 20 fs. For the DPPC membrane model, the time duration was set to 6.0 *μ*s and each system generated six parallel samples for analysis. The simulation time was elongated to 20 *μ*s for the lipid nanodomain model to capture sufficient configure samplings.

Martini 3.0 parameters: initial equilibration was carried out by performing energy minimization using the steepest descent algorithm, followed by a short MD run of 10 ps with the protein backbone beads restrained. Production runs were performed at 320 K using a velocity-rescale thermostat (44), with separate temperature coupling for the protein, lipids, and solvent particles. The pressure was maintained at 1 bar using a Parrinello-Rahman barostat (45), along with a semi-isotropic pressure coupling scheme. The nonbonded interactions were calculated by generating a pair-list using the Verlet scheme with a buffer tolerance of 0.005. The Coulombic terms were calculated using a reaction field with a cutoff distance of 1.2 nm. A cutoff scheme was used for the van der Waals terms with a cutoff distance of 1.2 nm. The Verlet cutoff scheme was used for the potential shift (46). The MD time step was set to 20 fs. For the DPPC membrane model, the time duration was set to 6.0 *μ*s and each system generated six parallel samples for analysis.

### Analysis

To quantify the effects of PIP2 and palmitoylation on the dimerization of CD44, the binding free energy was calculated using the umbrella sampling method (47). The potential of mean force (PMF) as a function of pulling distance between CD44 monomeric proteins was calculated to estimate the binding free energy. For each system, a representative and stable dimeric structure was selected from the crossing angle evolution as the initial conformation. Taking the connection line between the two protein chains as the reaction coordinate, one of the chains was fixed in position and the other chain was pulled in the X or Y directions. During the pulling process, the backbones of the mobile chain were restricted by a force of 800 kJ · mol^−1^ · nm^−2^, with a pulling rate of 10 nm/ns for 1000 ps. A total of 22–23 separate window configurations were created with a distance gradient of 0.25 nm. Each window was then equilibrated for 20 ns with a constant force of 1000 kJ · mol^−1^ · nm^−2^ in three dimensions, followed by a 2.0 *μ*s simulation production to generate well-overlapping configuration distributions. The WHAM method was used for unbiased umbrella potentials. The association energy was computed with the weighted histogram analysis method (48). Statistical errors were estimated with bootstrap analysis (49).

The distance evolvements between the TM domains and an average of six samples of the CD44 series (e.g., Figs. 1 *c* and S2 *a*) were calculated to reflect the homodimerization efficiency. A distance less than 1.0 nm was adopted to judge the establishment of a dimerization state (34,50). Histograms and error bars were used to describe the average distance and standard deviation. The TM crossing angles represented by the column diagram distribution (e.g., Fig. 1 *d*) were collected from all the six trajectories during which the CD44 dimerization was established. More details in generating the crossing angle can be seen in (51). The residue-contacted matrixes were produced by *gmx mdmat* to describe the predominant dimeric structures (e.g., Fig. S2 *c*) and the contacted matrix was generated from a typical simulation run where the angle evolvement was stable (e.g., Fig. S3). A cutoff distance of 0.9 nm was used to definite the interacting scale between backbone beads of the TM domain. The lipid-protein contact distribution of CD44 series (e.g., Fig. 5 *d*) was calculated using the *gmx mindist* tool, and a cutoff distance of 0.6 nm was used to definite the interacting scale between lipids and protein. Two-dimensional density maps were used to present the positions of CD44 series relative to the *Lo* subdomain. The protein was precentered and only DPPC lipids were taken into consideration to position the *Lo* subdomain (e.g., Fig. 6 *c*). Density map utilization centers of mass and the heatmaps were optimized by the software package Gnuplot-5.4.0 (http://www.gnuplot.info/).

## Results

### Dimerization of CD44 containing two juxta-membrane cysteines

The CD44 model used in the study, which consists of the TM domain and a truncated CT domain (Figs. 1, *a* and *b*), has been proved to be responsible for interacting with the cytoskeleton protein partner (14). The sequence contains two cysteine residues (Cys-286 and Cys-295) located at the juxta-membrane domain, such allows for exploring the interaction between the two pairs of cysteine as discussed previously (8). To characterize the dimerization of the CD44s plus the CT domains, two different lipid bilayer systems, consisting of either 100% DPPC or partially 5% PIP2, were recruited for simulation (Fig. S1 *a*). Two CD44-WT monomers were incorporated into the membrane with an initial distance of 5.88 nm (Fig. S1 *b*), and six independent replicates, each for a simulation time of 6.0 *μ*s (equals to an effective time of 24 *μ*s, considering an accepted speed-up factor of 4 in the Martini CG model compared with the experimental and atomistic simulations involving lipid lateral diffusion rates (34,51)) were used for analysis.

CD44-WT can rapidly form homodimer observed from six replicates, and the dimerization state maintains until the last 6.0 *μ*s (Figs. 1 *c* and S2 *a*). The TM crossing angle distribution shows that the dimeric conformation locates in a concentrated manner at −25° (Fig. 1 *d*), displaying as a stable right-handed (RH) mode. The residue contacted matrixes (Fig. S2 *c*) were used to unravel the packing details based on a typical simulation run where the angle evolvement was stable (Fig. S3). The dimer interface of CD44-WT is occupied by a packing motif of A274xxxA278xxxI282xxxC286 (Fig. 1 *d*). The packing alignment covers the 286^th^ cysteines that are reported to form a disulfide bridge. The distance between the 295^th^ cysteines at the CT domain is lower than the distance between the full-length CTDs (Fig. S4). The results are in agreement with existing experimental data that mutation of C295A retains a considerable dimerization capacity, while the C286AC295A can basically abrogate dimerization (8), implying the importance of the 286^th^ cysteine in CD44 dimerization (21). We note that the interaction between the 295^th^ cysteines is unstable throughout the simulations, which mostly results from the random folding of the CT domain in addition to the potential membrane attachment.

The latest open-beta 3.0 version was supplementary applied to predict the dimerization of CD44. The results show that CD44 can quickly form a homodimer in a similar case of 2.2 conditions, and it remains established until the last 6.0 *μ*s (Figs. 1 *d* and S2 *b*). The crossing angles of the packed TM domains remains at −25° RH conformation (Fig. 1 *e*), and the interaction of the 286^th^ cysteines also occur on the TM package interface (Fig. S2 *d*). The results under two force field versions thus reveal matchable packing information during the CD44 dimerization process.

### CD44 dimerization influenced by palmitoylated modifications

**Figure 2:**
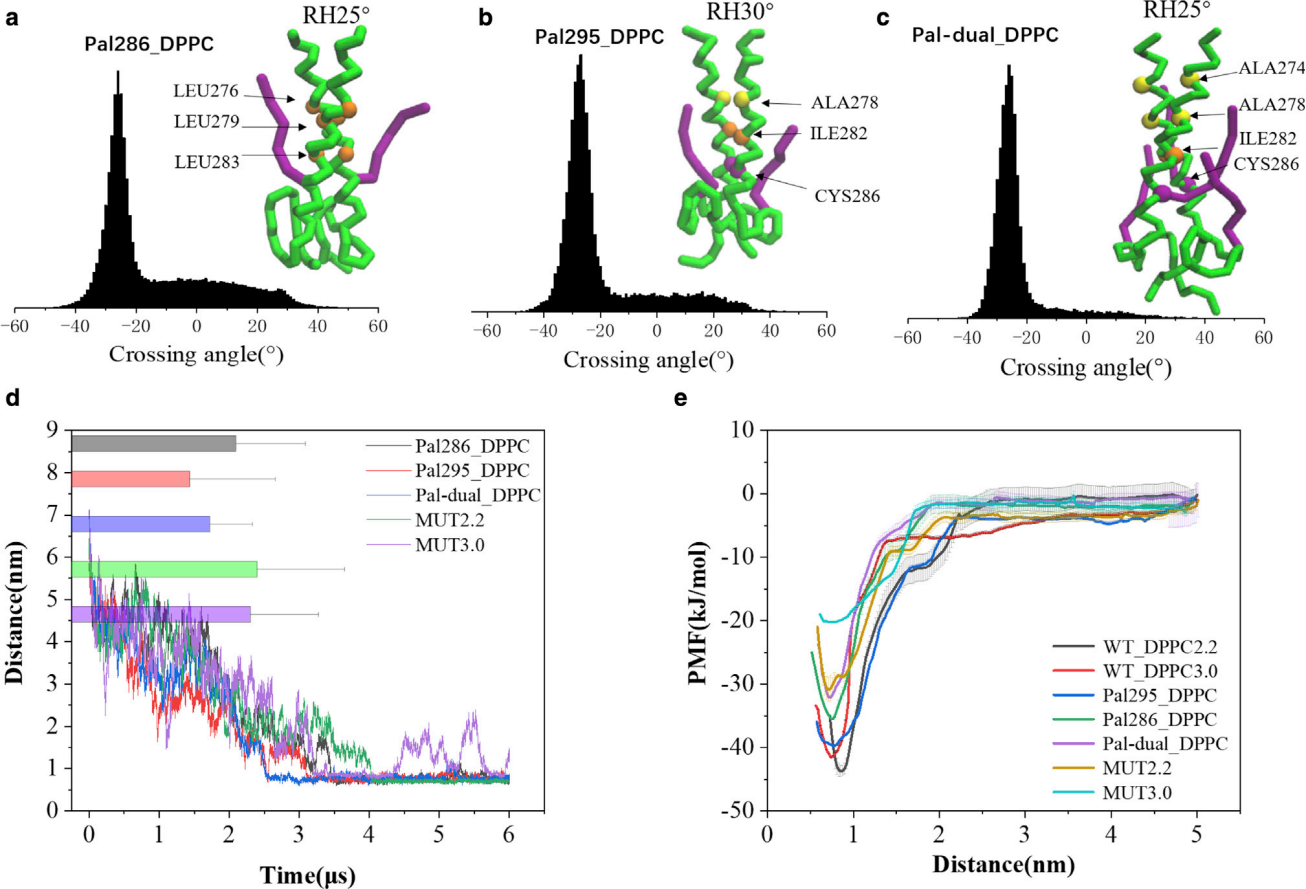
Dimerization and conformation of Pal-286, Pal-295, and Pal-dual in the pure DPPC bilayer. Presentations of the dimeric structure and the packing residues of Pal-286 (*a*), Pal-295 (*b*), and Pal-dual (*c*), respectively. (*d*) The distance evolvements between the TM domains averaged from six samples of the CD44 series. Histograms and error bars were used to describe the average distance and standard deviation. (*e*) The potential of mean force (PMF) of CD44 series by umbrella sampling method. More details can be seen in the Methods. To see this figure in color, go online.

To explore the influence of palmitoylation on CD44 dimerization, CD44 proteins modified with palmitoyl chains, at the 286^th^ cysteine (Pal-286), the 295^th^ cysteine (Pal-295), and both the 286^th^ and 295^th^ cysteine (Pal-dual), respectively, were subjected to simulations. The results show that the dimerization of six Pal-286 replicates is interfered by palmitoylation (Figs. 2 *d* and S5 *a*). Although the proteins in all the samples are finally assembled, it needs a longer time to complete dimerization. The weakened dimerization situation is also observed for Pal-295 (Figs. 2 *d* and S5 *b*) and Pal-dual (Figs. 2 *d* and S5 *e*). Analyzed from the dimeric conformation of Pal-295, the protein package exhibits a stable −30° RH conformation with a A278xxxI282xxxC286 motif on the interface, a consistent binding mode to the WT (Figs. 2 *b* and S6 *b*). In comparison, the Pal-286 exhibits a −25° RH conformation with L276xxxL279xxxL283 appearing on the interface instead (Figs. 2 *a* and S6 *a*), implying a structure reorientation of the TMD. The dimer of Pal-dual recovers as a stable −25° RH conformation packed by the A274xxxA278xxxI282xxxC286 motif on the interface (Figs. 2 *c* and S6 *c*), mostly derived from a combining reorientation effect from the two palmitoyl chains. To further verify the positive effect of the two cysteines on CD44 dimerization, a mutant of C286AC295A was subjected to simulations in 2.2 and 3.0 versions. It can be observed that abrogation of the two cysteines cause decelerated dimerization of CD44 (Fig. 2 *d*). The results thus the reveal negative influence of the palmitoylated modifications on CD44 dimerization, which is dependent more on the cysteine at the TM domain.

To further quantify the effect of palmitoylation on CD44 self-association, the PMF of the palmitoylated CD44 series was calculated using the umbrella sampling technique. The energy cost for completely dissociating the dimer of CD44-WT in Martini 2.2 is 44 (±0.4) kJ/mol (Fig. 2 *e*). In contrast, it deceases to 36 (±0.35), 40 (±0.21), and 32 (±0.73) kJ/mol for the Pal-286, Pal-295, and Pal-dual, respectively. The reduced energy barrier in disassociating the palmitoylated dimer is therefore in agreement with the weakened dimerization as discussed above. Correspondingly, the energy cost of the Pal-286 is lower than the Pal-295, indicating the predominant role of the TM interaction in CD44 dimerization. The dual palmitoylation shows a minimum energy cost, suggesting a combining effect of the double palmitoylation. Under the Martini 3.0 condition, it costs 42 (±0.24) kJ/mol to dissociate the CD44-WT. This matches to the overestimated dimerization propensity in Martini 2.2 as discussed, but the energy estimation behaves at a comparable level with the 3.0 version. The energy cost of the C286AC295A in Martini 2.2 and 3.0 is reduced to 31 (±079) and 20 (±0.52) kJ/mol, respectively (Fig. 2 *e*), which reflects an increased monomerization propensity after a cysteine removal. Taken together, these results demonstrate the importance of cysteine in the juxta-membrane regions, and an insertion of the palmitoyl chain is unfavorable for CD44 self-recognition.

### Dimerization of CD44 palmitoylated variants promoted by PIP2

**Figure 3:**
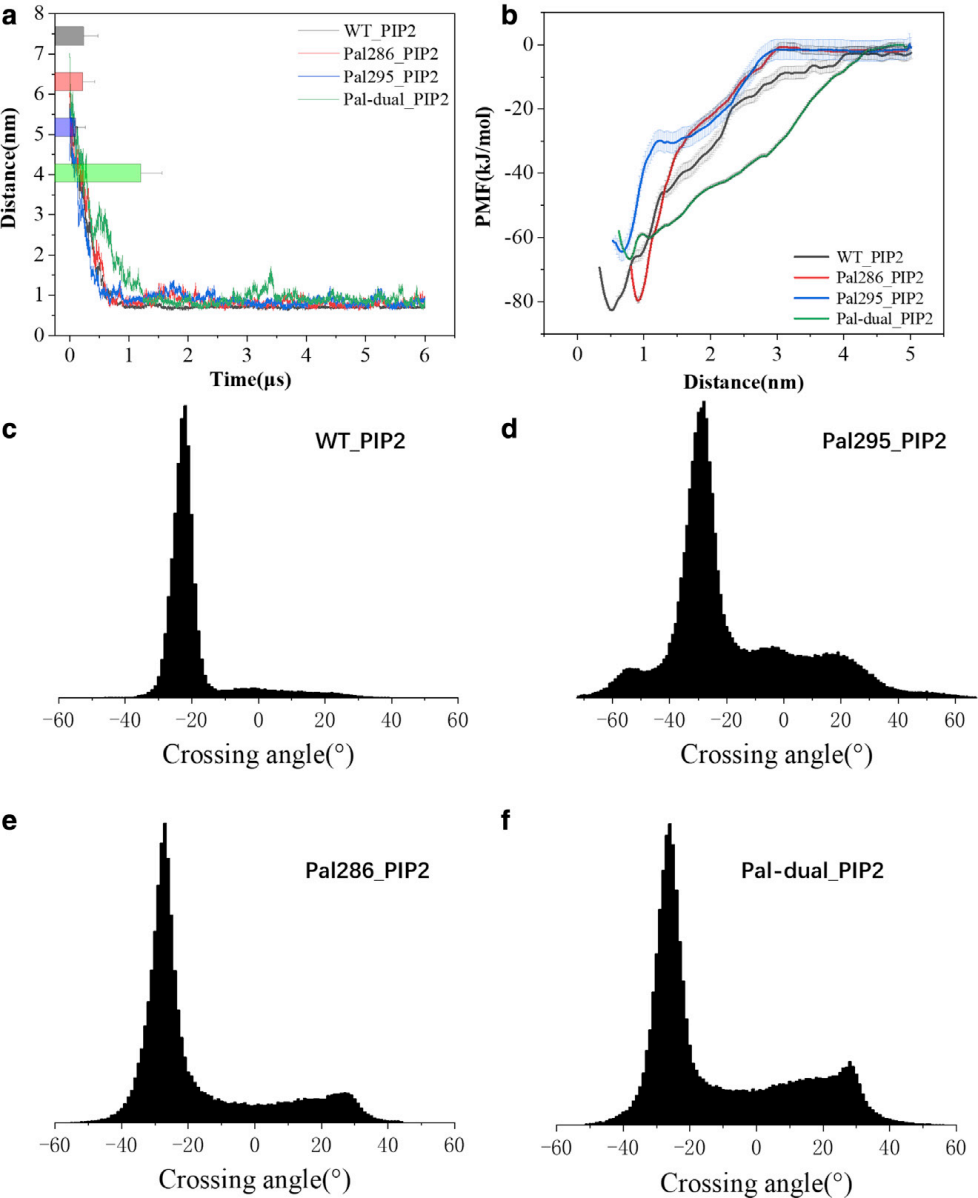
Dimerization and conformation of the CD44-WT, Pal-286, Pal-295, and Pal-dual in the DPPC bilayer containing PIP2. (*a*) The distance evolvements between the TM domains averaged from six samples of the CD44 series. Histograms and error bars were used to describe the average distance and standard deviation. (*b*) The PMF of CD44-WT, Pal-286, Pal-295, and Pal-dual. Crossing angle distribution of the dimeric (*c*) CD44-WT, (*d*) Pal-295, (*e*) Pal-286, and (*f*) Pal-dual. To see this figure in color, go online.

Multivalent anionic PIP2 has been found to mediate CD44 assembly in solution, but the regulation philosophy remains unclear for the case of CD44 dimerization influenced by palmitoylated modifications and PIP2. For this purpose, PIP2 was incorporated into the lower membrane leaflet with a mole percentage of 5% (Fig. S1 *a*; Table S1). Dimerization of CD44-WT establishes rapidly in the presence of PIP2 (Figs. 3 *a* and S7 *b*), in accordance with existing data that PIP2 is favorable for CD44 clustering. It is noteworthy that the palmitoylated CD44 series showing defected dimerization efficiency can also realize dimerization within 1.0 *μ*s observed from all the replicates. However, the distance evolvements of the palmitoylated CD44 series exhibit evident fluctuation, especially of the Pal-295 and Pal-dual (Fig. S7). The phenomenon implies that the dimeric packages of CD44 under palmitoylation remain unstable, although there is an association driving by PIP2 molecules.

**Figure 4:**
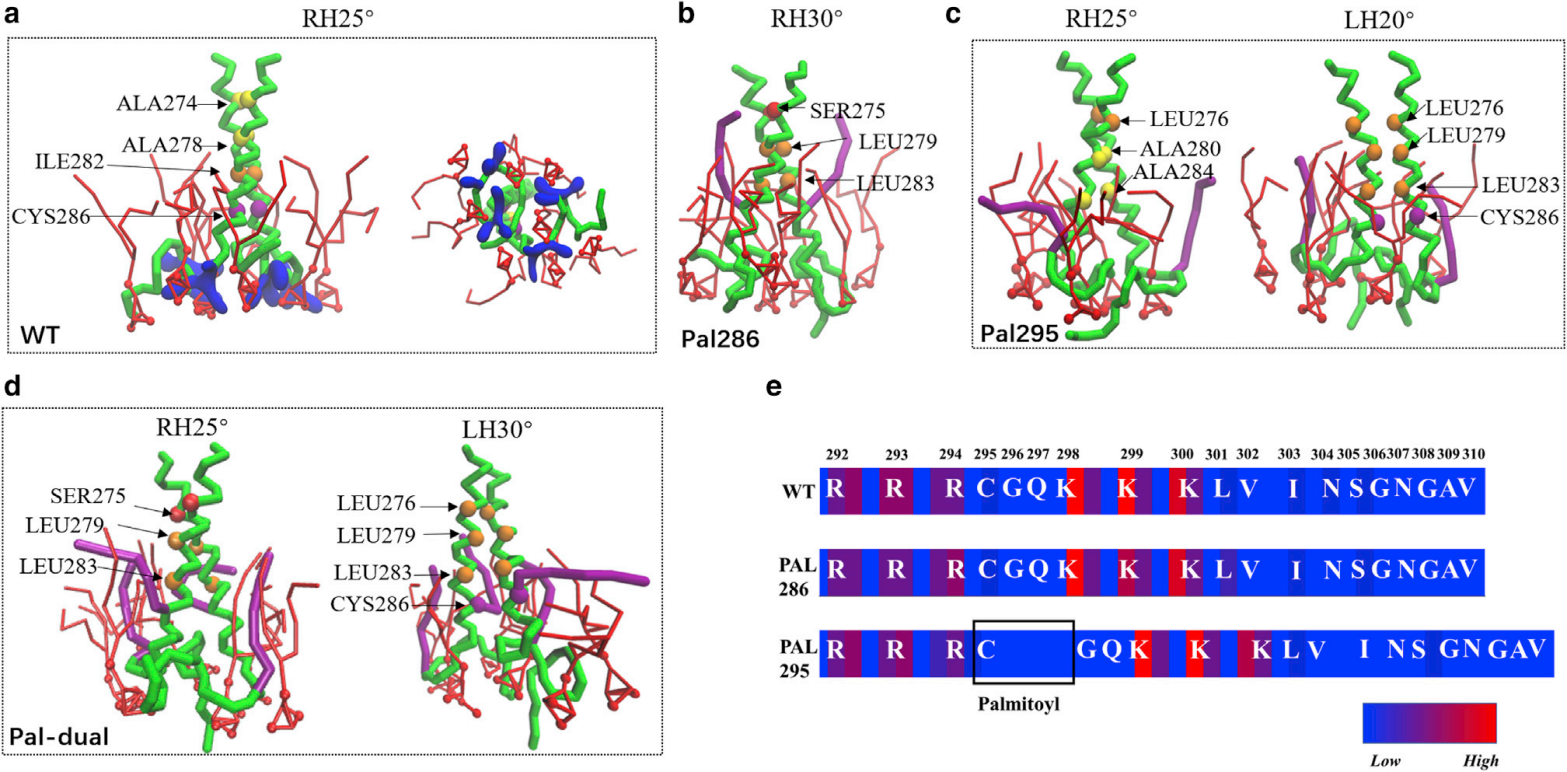
Dimerization details of the CD44-WT, Pal-286, Pal-295, and Pal-dual in the DPPC bilayer containing PIP2. Presentations of dimeric structures of (*a*) CD44-WT, (*b*) Pal-286, (*c*) Pal-295, and (*d*) Pal-dual. PIP2 is shown in red, R292-294 and K299-301 are shown in blue. (*e*) The contact maps of CD44 residues in binding to PIP2. Only the time frames during which the distance between residues (292 to 310) on the CT domain and PIP2 was less than 0.5 nm were counted. The residue occurring frequency in all the selected frames was analyzed to generate the contact hot maps. To see this figure in color, go online.

In the presence of PIP2, the dimer of CD44-WT remains as a stable −25° RH conformation packed by the A274xxxA278xxxI282xxxC286 motif on the interface (Figs. 3 *c*, 4 *a*, and S7 *a*). In contrast, the crossing angle distribution of Pal-286 shifts to a predominant −30° RH mode with a S275xxxL279xxxL283 motif on the interface (Figs. 3 *e*, 4 *b*, and S7 *e*), while an extensive distribution to the left-handed (LH) mode of 30° occurs. The conformation switching becomes more severe for Pal-295, with the dimeric structure varying from t −25° RH to 20° LH, and the predominant contacted residues also change to L276xxxA280xxxA284 and L276xxxL279xxxL283xxxC286, respectively (Figs. 3 *d*, 4 *c*, and S7 *c*). Similarly, the protein package of Pal-dual exhibited conformations of −25° RH and 30° LH with S275xxx L279xxx L283 and L276xxxL279xxxL283xxxC286 and motif on the interface, respectively (Figs. 3 *f*, 4 *d*, and S7 *g*). The results are therefore consistent with the distance evolvements with strong noises (Fig. 3 *a*), reflecting a susceptive dimerization of CD44 regulated by palmitoylation and PIP2 addition. The structural feature is further confirmed by the larger gap between the TM-C-terminals of Pal-286 (1.39 nm) and Pal-dual (2.01 nm) than that of CD44-WT (1.14 nm) (Fig. S8).

The PMFs were calculated to quantify the dimerization ability affected by palmitoylation and PIP2 (Fig. 3 *b*). Compared with the pure DPPC systems, the energy cost for dissociating the dimers of CD44-WT, Pal-286, Pal-295, and Pal-dual increases to 84 (±1.73), 80 (±1.23), 64 (±3.13), and 67 (±0.46) kJ/mol, respectively. The results indicate a stronger protein package mediated by PIP2, which is consistent with the rapid TM distance evolvements above. The well depth of PMFs of Pal-295 and Pal-dual is much lower than CD44-WT and Pal-286, which corresponds to the more severe dimeric structural fluctuation of Pal-295 in the presence of PIP2 (Fig. 3, *d* and f; Fig. 4, *c* and *d*).

It is inferred that the 295-palmitoyl on the CT domain impedes the adhesive interaction of PIP2 and protein, leading to a switching binding conformation of Pal-295. Protein-PIP2 binding details were subsequently analyzed to explore the regulation mechanism of PIP2. The results show that, consistent among CD44-WT, Pal-286, and Pal-295, PIP2 is found to mainly interact with the basic residues of R292-294 and K299-301 in the intracellular domain (Fig. 4 *e*). Because the palmitoyl chain on the Cys-295 locates correctly between the two PIP2 adhering motifs, it is conceivable that an interference of the palmitoyl group happens for the PIP2-mediated protein dimerization. As a consequence, the conformation of Pal-295 is more chaotic and appears as multiple reconstructions.

### CD44 dimerization mediated by lipid raft and PIP2

**Figure 5:**
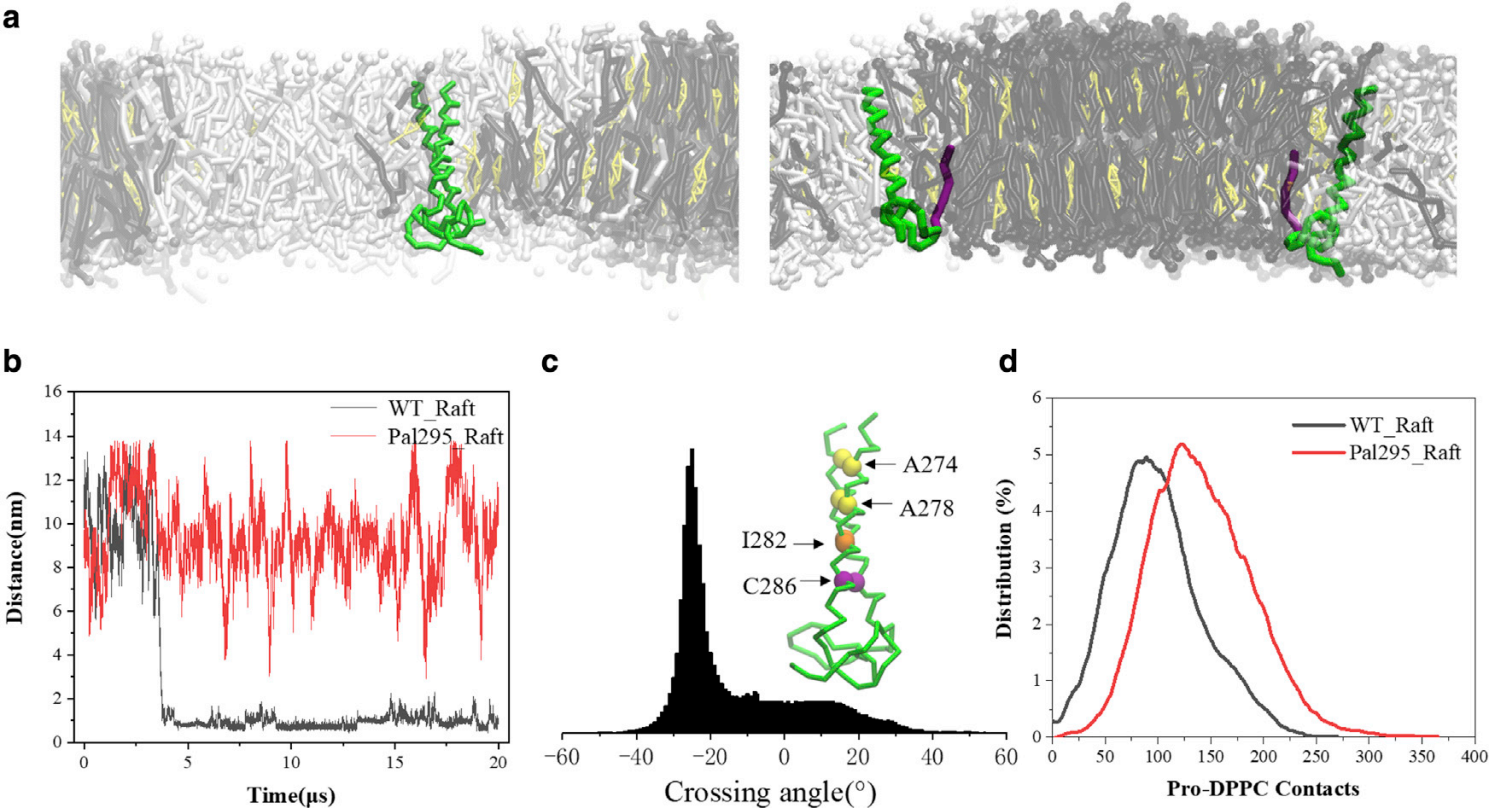
Comparisons of the dimerization and localization of CD44-WT and Pal-295 regulated by lipid raft. (*a*) Localization and structure of CD44-WT (*left panel*) and Pal-295 (*right panel*) in the binary phase membrane. DPPC, DIPC, and cholesterol are presented as black, white, and yellow, respectively. (*b*) The distance evolvements between the TM domains of CD44-WT and Pal-295, respectively. (*c*) The crossing angle distribution of WT-CD44 and the dimeric structure presentation. (*d*) The DPPC-protein contact distributions of CD44-WT and Pal-295, respectively. To see this figure in color, go online.

The lipid raft has been found to regulate the translocation and acceptor recognition of CD44, acting as a crucial linkage between protein palmitoylation and cell migration. To explore the effect of lipid nanodomain on the dimerization of CD44 under different palmitoylations, the bilayer model composed of DPPC/DIPC/CHOL (40:40:20) used in our previous work (14) is employed herein (Fig. 5 *a*; Table S1). The lipids randomly distributed initially and they segregated into an apparent binary phase (*Lo* subdomain and *Ld* subdomain) bilayer within 1.0 *μ*s. Dimerization of CD44-WT can basically establish after 3.7 *μ*s (Fig. 5, *a* and *b*), except for a somewhat loose package after about 13 *μ*s (Figs. 5 *b* and S9 *a*). The protein package of CD44-WT exhibited a predominantly −25° RH conformation packed by the motif of A274xxxA278xxxI282xxxC286 (Figs. 5 *c* and S9 *a*), in agreement with that in pure DPPC membrane. We note that the dimeric CD44-WT remains to partition in the *Lo* subdomain (raft affiliation) to a large extent (Fig. S10 *a*), whereas the CD44-WT monomer mainly partitions in the *Ld* subdomain (14). Raft affinity of TM proteins enhanced by oligomerization was also found in a previous study (13).

To investigate the influence of palmitoylation, Pal-295 was recruited in simulations on account of the known stronger raft affiliation of 295-palmitoyl (14). In contrast, no dimerization was observed for Pal-295 throughout the 20 *μ*s simulation timescale (Fig. 5 *b*). This suggests that the binary phase bilayer exerts a more severe prohibition on Pal-295 dimerization than that in the single-phase bilayer (Fig. 2 *d*). By analyzing the contact intensity of protein with DPPC lipids (raft marker), Pal-295 is found to show a higher raft affiliation compared with CD44-WT (Fig. 5 *d*). This characteristic was further verified by the two-dimensional density distributions of two monomers of Pal-295 that attach at the raft boundary (Fig. S10 *b*). The higher raft affinity of Pal-295 is attributed to the raft anchoring of the palmitoyl chain (Fig. 5 *a*), which abolishes the ability of peptides to move freely and then dimerize. To compare the dimeric ability by palmitoylation affected by different membrane phases, two CD44-WT monomers were preplaced in the *Lo* subdomain, while two Pal-295 monomers were preplaced in the *Ld* subdomain (Fig. S11). Influenced by the low fluidity of the *Lo* phase, the WT cannot be aggregated throughout a 10 *μ*s duration. Pal-295 maintains a separated state for a marked *Lo* infinity. These results prove that CD44 dimerization is inherently interrupted by the raft localization propensity, which is motivated by palmitoylated modifications. Our previous study also demonstrated that PIP2 could compete with the *Lo* subdomain to guide TM protein translocation between membrane subdomains (14).

**Figure 6:**
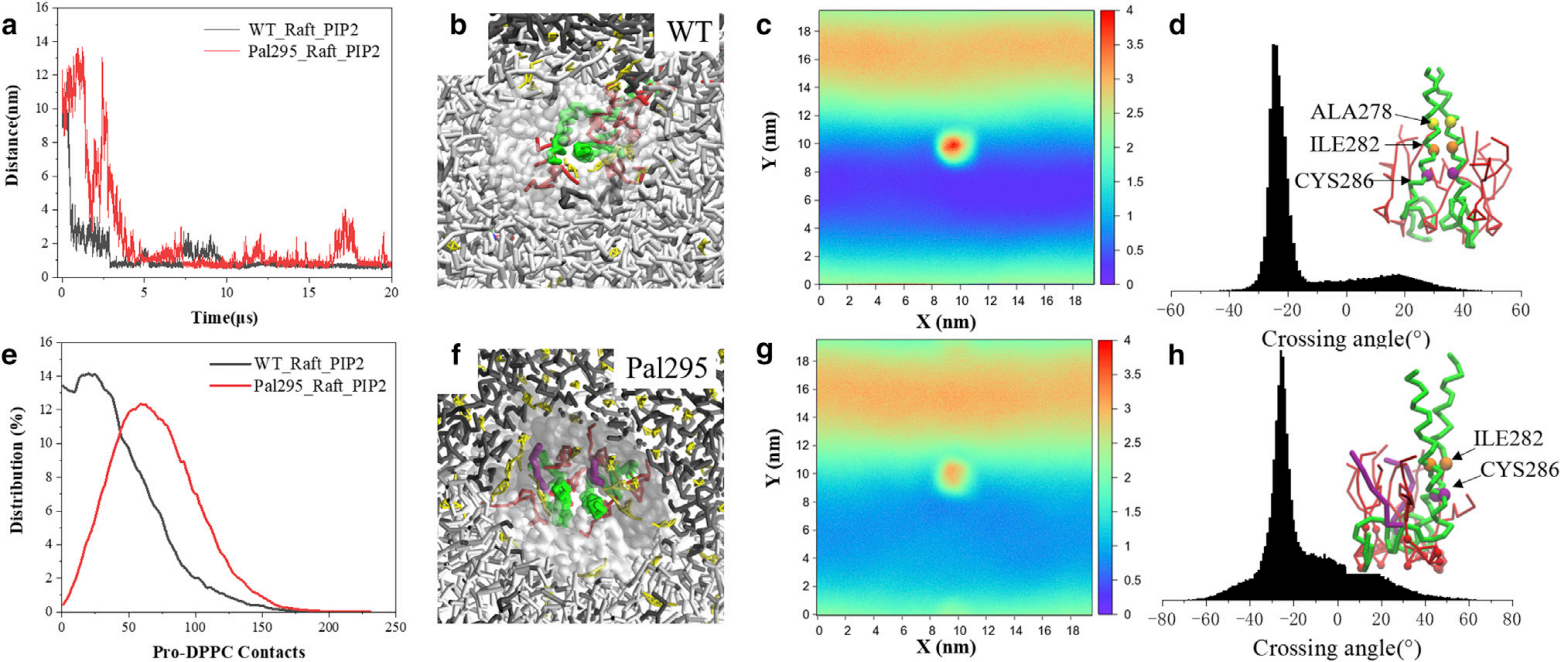
Comparisons of the dimerization and localization of the CD44-WT and Pal-295 in the segregated bilayer with PIP2 inclusion. (*a*) The distance evolvements between the TM domains of CD44-WT and Pal-295, respectively. (*b*) Localization and dimeric orientation of CD44-WT and (*f*) Pal-295 in the binary phase membrane. (*c*) Two-dimensional density maps of CD44-WT and (*g*) Pal-295 relative to the *Lo* subdomain marked by high density region. The TM crossing angle distribution and presentations of the dimeric structures of (*d*) CD44-WT and (*h*) Pal-295, respectively. (*e*) The DPPC-protein contact distributions of CD44-WT and Pal-295, respectively. To see this figure in color, go online.

To determine the effects of PIP2 on CD44 dimerization, PIP2 molecules with a mole percentage of 2% (Table S1) were incorporated into the lower leaflet, with all the lipid components distributed randomly at the simulation start. Different from the system without PIP2 (Fig. 5 *b*), both CD44-WT and Pal-295 can associate into homodimers, although Pal-295 requires nearly 4.5 *μ*s (Fig. 6 *a*). The results imply that PIP2 remains efficient in TM protein packing in the phase-segregated bilayer. A snapshot of dimeric CD44-WT surrounded by PIP2 molecules is shown in Fig. 6 *b*. It is evident that CD44-WT inclines to reside in the *Ld* subdomain, which is further proved by a two-dimensional density map (Fig. 6 *c*). The phenomenon is also confirmed by the weakened DPPC contact of the WT as well as Pal-295 when PIP2 is included (Fig. 6 *e*). It is observed that the dimer of Pal-295 prefers to reside loosely at the boundary of the *Lo* subdomain (Fig. 6, *f* and *g*). Collectively, these results reveal the exclusive function of PIP2 in releasing the palmitoylated CD44 from the *Lo* subdomain and, further, prompts protein to dimerize. In fact, the raft-interacting orientation of the palmitoyl chain reflects a competition relationship between PIP2 and palmitoyl in determining protein position (Fig. 6 *f*). The results indicate that PIP2 adhering on protein can greatly release the position immobility of protein by palmitoylation, which provides increased possibility for CD44 in linkage with its cytoskeleton adaptor in the binary phase membrane.

In the presence of PIP2, the dimerization of CD44-WT is packed by a A278xxxI282xxxC286 motif, and it exhibits a more stable −25° RH conformation than that in the absence of PIP2 (Figs. 6 *d* and S9 *b*). The packing mode is basically consistent with the situations without phase segregation as discussed above. Note that, although Pal-295 realizes dimerization modulated by PIP2, the distribution of crossing angle is more disordered, consistent with the cases found in the single-phase membrane (Fig. 3). The predominant residue contact changes to a C-terminal contact of I282xxxC286 (Figs. 6 *h* and S9 *c*) with the cytoplasmic tail length getting increased (Fig. S12). The structural character has been proposed as a precondition for associating with the cytoskeleton linker proteins (14). In other words, the PIP2-mediated protein oligomerization in the raft-forming bilayer becomes susceptive in structure, and the property is correlative with the processing of cell proliferation.

## Discussion

Acylated modification and specific membrane environments are of importance for protein translocation and association, controlling many biological processes, such as protein clustering (52) and cell migration (53). CD44 is expressed in many tissue cells in humans and is overexpressed in cancers (54). Cleavage of the ETD of CD44 promotes tumor cell metastasis and invasion, which is regulated by the lipid modification of the CT domain and linkage of ERM or merlin with CD44 dimers. In breast cancer tumor cells, CD44 modified by palmitoylation is considered to prevent cell migration, so a blocking of palmitoylation on the transmembrane proteins acts as a potential approach to inhibit tumors (14,55,56). Our results prove that palmitoylation is unfavorable for CD44 dimerization and disturb the dimeric configurations, which helps stablish the linkage between post-transduction modification and cell irregular proliferation. This study reveals that PIP2 promotes the dimerization of CD44 under different palmitoylation states, which is favorable for tumor cell metastasis. This is consistent with previous studies that the association of CD44 and FERM is improved by PIP2 (14,22), a trigger in tumor cell invasion (57).

CD44 predominantly form a stable RH homodimer packed by the TM domains varied with lipid compositions. Although the disulfide bridge on cysteine residues cannot be spontaneously built in the CG condition, the dimeric interfaces show closely interrelation of the 286^th^ cysteines, together with other residues like A274xxxA278xxxI282, with CD44 assembly. It is noteworthy that the suppressed effects of Pal-286 and Pal-295 on dimerization are discrepant. Palmitoylation on the 286^th^ cysteine remarkably weakens CD44 dimerization. It is consistent with that the TM domain is more difficult to be palmitoylated by palmitoyl acetyl-transferases (58,59), therefore CD44 exists as homodimers in the resting state. In contrast, the palmitoylation on the cytoplasmic 295^th^ cysteine residue is more accessible. It seems indispensable as the Pal-295 is more favorable for CD44 approaching the lipid raft (14), thus reducing the dimerization ability. This provides a necessary explanation for the lower dimerization decrease by blocking the 295^th^ cysteines, since the interaction between them is not sufficiently strong (11). At the same time, mutating-specific amino acid sites not only weaken the ability of CD44 to dimerize but also cause it to produce an unstable conformation, corresponding to the biological expression of transmembrane proteins, which is often directly affected by the transformation of their structures.

Note that we used one-dimensional PMF initiated from a bound state to quantify the dimerization ability of CD44 palmitoylated variants, and the energy convergence near the first well depth has been proven efficient and changes slightly with the simulation timescale (60, 61, 62, 63), thus allowing for a significant comparison for dimerization ability of TM protein affected by mutations or chemical modifications. The PMF from an unbound state is not adopted on account of the slow efficiency and occurrence of nonnative bound events (60). It is known that increasing simulation sampling is required for a good accuracy of the remaining shallow well depth on PMF tendency. To compensate for the reduced PMF convergence in this study, we paid sufficient attentions to the occurrence of switching crossing angle distribution and the relevant interface details to provide full insights into the dimerization difference of CD44 affected by membrane environments and palmitoylation. Two-dimensional PMF calculation showing the relevance of TM crossing angle and rotation around the protein axis is good for assembly of proteins merely containing TMDs (64,65), whereas it seems a challenge in computation for the TMD tethered by a CTD (like CD44 herein), as an extra rotation variable of the CTD around the TMD (0–360°) should be counted. Considering the asymmetry of two protein monomers, it eventually generates millions of configuration windows for simulation, causing the computation burden to be too heavy. This study took into full consideration the dimerization efficiency, the TM crossing angle information from all dimerization phase, and the binding free energy to elaborate the changeable dimerization affected by palmitoylations and membrane environments. To compensate an over-estimated self-assembly of TM proteins in CG condition, high-throughput sampling approaches, such as temperature replica exchange molecular dynamics, are encouraged to obtain more statistical dissociation rate.

Lipid rafts are known to act as an organization platform for various transmembrane proteins. In breast cancer tumor cells, raft localization of palmitoylated CD44 is considered to prohibit cell migration (14). In line with experimental results, we found that palmitoylation on the 295^th^ cysteine shows higher raft affiliation compared with CD44-WT (14). The higher raft affinity of Pal-295 abolishes the ability of the protein to move freely and dimerize. As a consequence, palmitoylation plays an increased inhibitory role in protein dimerization mediated by lipid nanodomains (Fig. 5 *d*). Because the palmitoylated protein finds it difficult to completely enter the *Lo* subdomain (lipid raft) under the current simulation condition, it is hard to judge whether a dimerization will form in the lipid raft. A recent study has revealed that CD44 exists as homodimers in tumor cells rather than in normal cells. Combining with the upshifted association of CD44 and FERM in the nonraft, a stimulator of tumor cell migration, it is surmised that CD44s modified by palmitoylation exist as monomers when approaching the lipid raft. PIP2 addition is efficient for the dimerization of CD44 palmitoylated either at Cys-285 or Cys-295. It compensates for the defected dimerization of Pal-295 by a release from the lipid raft, in line with our previous data that PIP2 competes with the lipid raft to guide TM protein translocation between membrane subdomains. However, their dimeric configurations, especially of Pal-295, are less stable compared with the WT. One explanation for this is that the palmitoyl chain locates between two PIP2-binding motifs (R292-294 and K299-301) (Fig. 4 *e*), so a remarkable interference takes place when PIP2 adheres to CD44. On the one hand, the phenomenon appears necessary for transferring the signal along the TM region as the rotational CT dimeric configuration is proposed to correspond to the inside-out signal transduction of CD44 (8). On the other hand, the changeable dimeric conformation of CD44 affected by modification and membrane environments is related to the release of CTD to activate the downstream gene expression, which is implicated with tumor cell growth and proliferation (9). For CD44-WT dimerization in the PIP2-included phase-segregated bilayer, a transformational dimerization occurs along with a changeable arrangement of CT domains (Fig. S13), in consistence with the monomer found in our recent work (14). Taken together, the changeable configuration of the CT domains regulated by specific membrane environments acts as a precondition in association with the cytoskeleton linker proteins and mediates cleavage of the ET domain.

**Figure 7:**
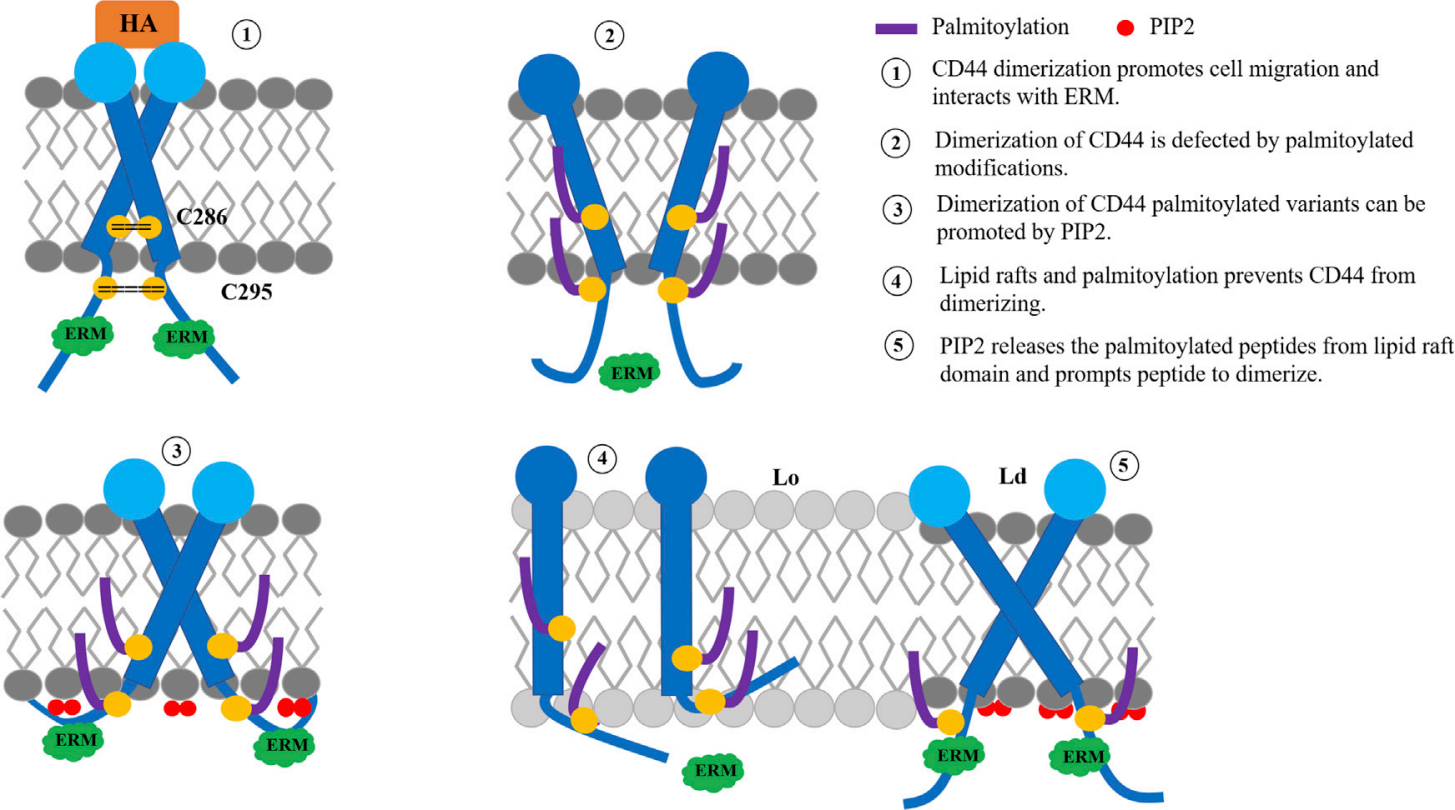
Schematic representation for the molecular mechanism of CD44 homodimerization modulated by palmitoylations and PIP2 in different membrane environments. To see this figure in color, go online.

It is inferred that homodimerization and localization of CD44 is sensitive to palmitoylation and specific membrane environments (Fig. 7). Firstly, the homodimerization of WT is dependent on the interaction of cysteines on the juxta-membrane domain, which is favorable for HA binding of the ET domain. An insertion of a palmitoyl chain is unfavorable for CD44 dimerization, because it prohibits the binding of CD44 to the cytoskeleton linker of ERM. Secondly, dimerization of CD44 palmitoylated variants can be promoted by PIP2 addition. The presence of PIP2 lipids allows ERM to bind to the surface of the membrane (21) and further benefits protein association. Thirdly, PIP2 lipids enable the palmitoylated CD44 that conceals in raft as monomers to migrate backward the nonraft domain, thus allowing for resuming dimerization and protein recognition. Due to transformation of the dimerization and localization, more signaling pathways by other bilayer/protein stimulators seem indispensable for CD44 in mediating cell proliferation and tumor progression.

Because of heterogeneous membrane microenvironments and modifications of proteins in different positions, the dimerization and localization of CD44 can modulated in multiple ways. It is inferred that a delicate balance exists between the homodimers and monomers of CD44 regulated by palmitoylation to different extents. Moreover, the location preference and dimeric conformation are also sensitive to specific lipids and palmitoylation. The resulting dimeric conformation may restrict the subsequent signal transduction process. Taken together, these results can expand our understanding of TM protein self-assembly modified by palmitoylation and membrane environments, and shed light on the regulation mechanism underlying the inside-out signal transduction of CD44-like proteins.

## Conclusions

In summary, we used CG dynamic simulations to explore the dimerization, localization preference, and structural deformation of CD44 in molecular detail. The results of this study reveal the molecular details of CD44 homodimerization in different membrane environments regulated by palmitoylation and PIP2 lipids on the one hand, and explain the regulation mechanism of palmitoylation as well as specific membrane positioning on the other hand. The dynamic molecular information reveals a delicate balance of protein palmitoylation and membrane composition, which strongly affects dimerization of CD44 and translocation direction. Given the sequence homology of single-spanning cell adhesion proteins accessed by palmitoylation at the juxta-membrane domain, the molecular inspection in this study is of broad significance for understanding biological function of CD44-like proteins.

## Author contributions

F.S., H.A., and Z.M. designed the research. F.S., Z.M., and M.R. performed the simulations. F.S. and C.P. contributed the analytic methods. Z.M., S.S. and Y.Z. analyzed the simulation results. Z.M., F.S., and H.A. wrote the article.

## Supporting material

Document S1. Figures S1–S13 and Table S1

Document S2. Article plus supporting material
