# Supplementary material for: Molecular mechanism of CD44 homodimerization modulated by palmitoylation and membrane environments
Source: Biophys J. 2022 Jun 22;121(14):2671–83. doi: 10.1016/j.bpj.2022.06.021 (PMC9382338; doi:10.1016/j.bpj.2022.06.021)
Supplement: Document S1. Figures S1–S13 and Table S1 [file mmc1.pdf]

Biophysical Journal, Volume *121*

Supplemental information

Molecular mechanism of CD44 homodimerization modulated by palmitoylation and membrane environments

Ziyi Ma, Sai Shi, Meina Ren, Chunli Pang, Yong Zhan, Hailong An, and Fude Sun

Supporting information

Molecular Mechanism of CD44 Homodimerization Modulated by Palmitoylation and Membrane Environments

Ziyi Ma[1], Sai Shi[1, 2, 3], Meina Ren[1], Chunli Pang[1], Yong Zhan[1, 2, 3], Hailong An[1, 2, 3]*, Fude Sun[1]*

[1] Key Laboratory of Molecular Biophysics, Hebei Province, Institute of Biophysics, School of Health Science & Biomedical Engineering, Hebei University of Technology, Tianjin, 300401, China

[2] State Key Laboratory of Reliability and Intelligence of Electrical Equipment, Hebei University of Technology, Tianjin 300401, China

[3] Key Laboratory of Electromagnetic Field and Electrical Apparatus Reliability of Hebei Province, Hebei University of Technology, Tianjin 300401, China

* Corresponding authors: sunfd@hebut.edu.cn; hailong_an@hebut.edu.cn.

Supporting Figures:

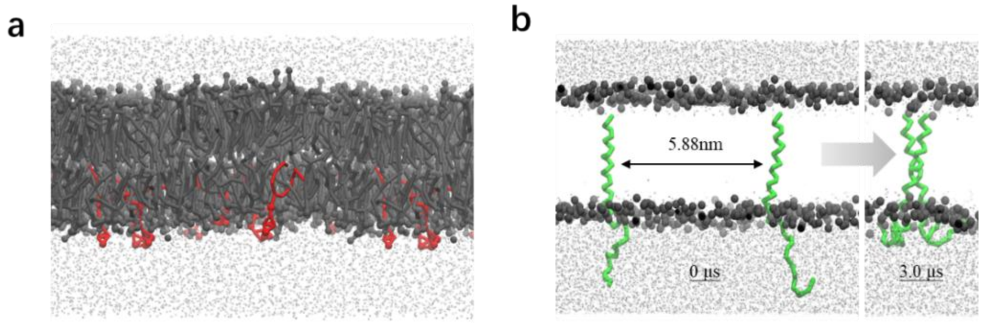

FIGURE S1. (a) CG lipid bilayer models consist of 95% DPPC and 5% PIP2. DPPC and PIP2 are shown in gray and red respectively, while the CG waters are shown in cyan. (b) Presentation of the protein-bilayer model at initial state and assembling state. Only the backbone beads of CD44 are shown. For clarification, the hydrophobic region of bilayer was omitted.

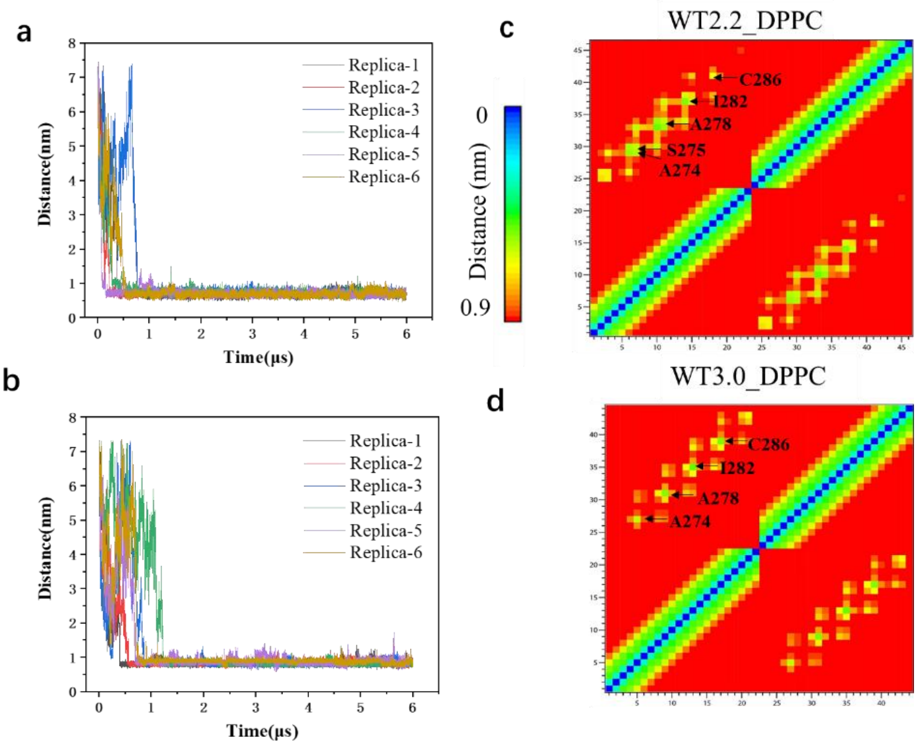

FIGURE S2. (a) Distance evolvements between the TM domains of CD44-WT in 2.2 version and (b) in 3.0 version. Six

independent runs were counted, respectively. (c) and (d) The residues contacted matrixes of the predominant dimeric structures in the DPPC membranes under two force field conditions. The data was generated from a typical simulation run where the angle evolvement is stable (Fig. S3). A cut-off distance of 0.9 nm was used to definite the interacting scale between backbone beads of TM domain.

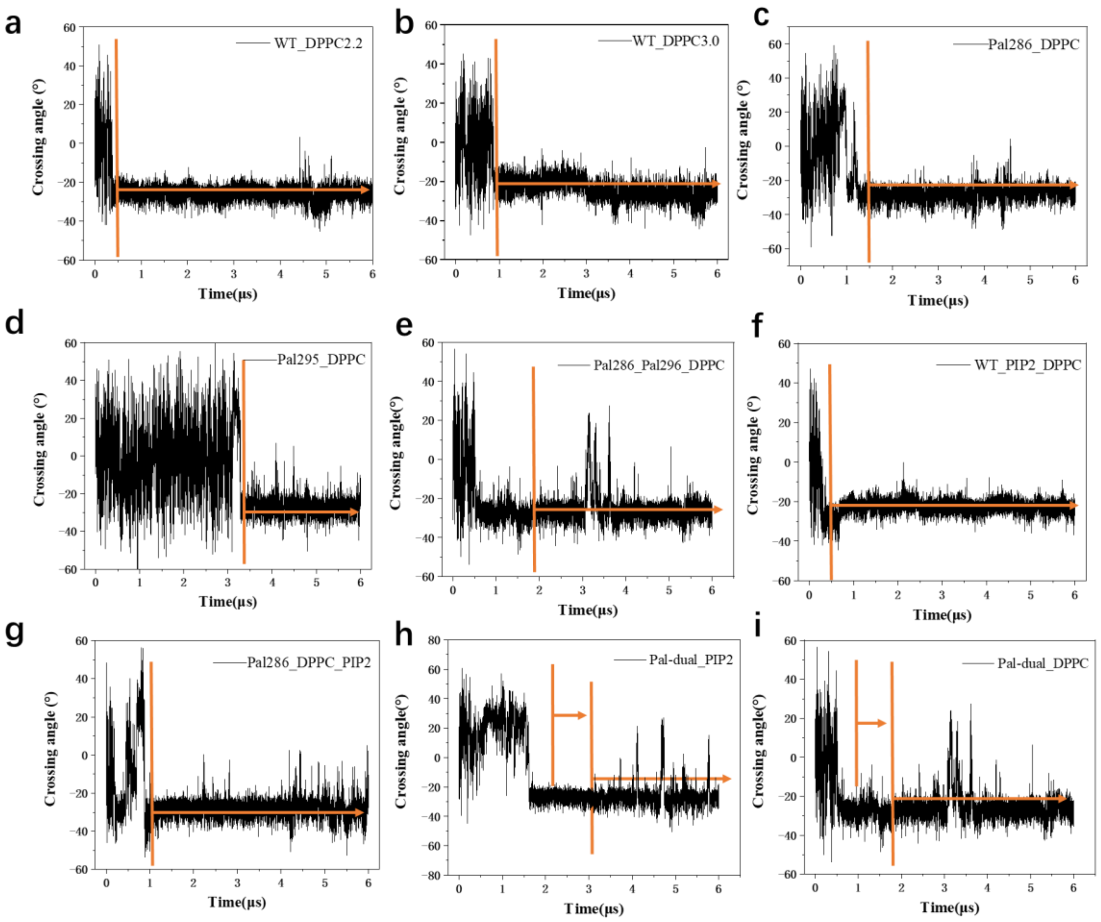

FIGURE S3. (a)- (i) TM crossing angle evolvements between the TM domains of CD44 series. Lines were added to indicate the dimerization-established state.

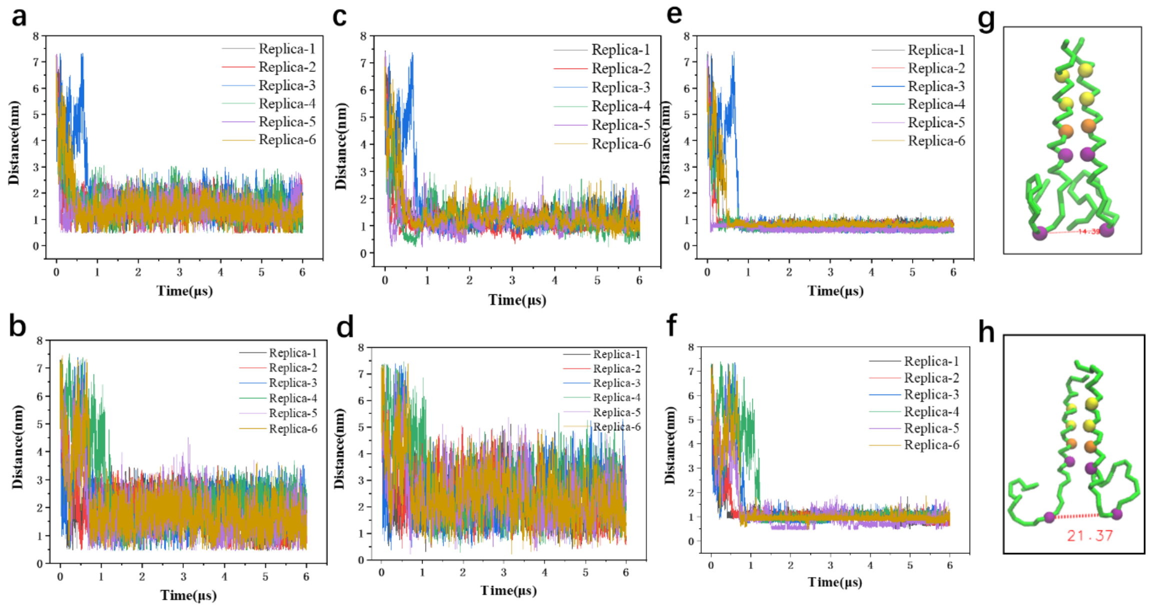

FIGURE S4. (a) Distance evolvements between backbones of 295th cysteines of CD44-WT in Martini 2.2 force filed and (b) in Martini 3.0 force filed. (c) Distance evolvements between the CT domains of the Martini 2.2 force filed and (d) in Martini 3.0 force filed. (e) Distance evolvements between backbones of 286th cysteines of CD44-WT in Martini 2.2 force filed and (f)

in Martini 3.0 force filed. (g) and (h) One snapshot of the dimeric CD44-WT in the pure DPPC bilayer. The distance between the backbones of 295th cysteines is shown, which reflects the weak bound state of the 295th cysteines. The upper panel is version 2.2. The lower panel is version 3.0.

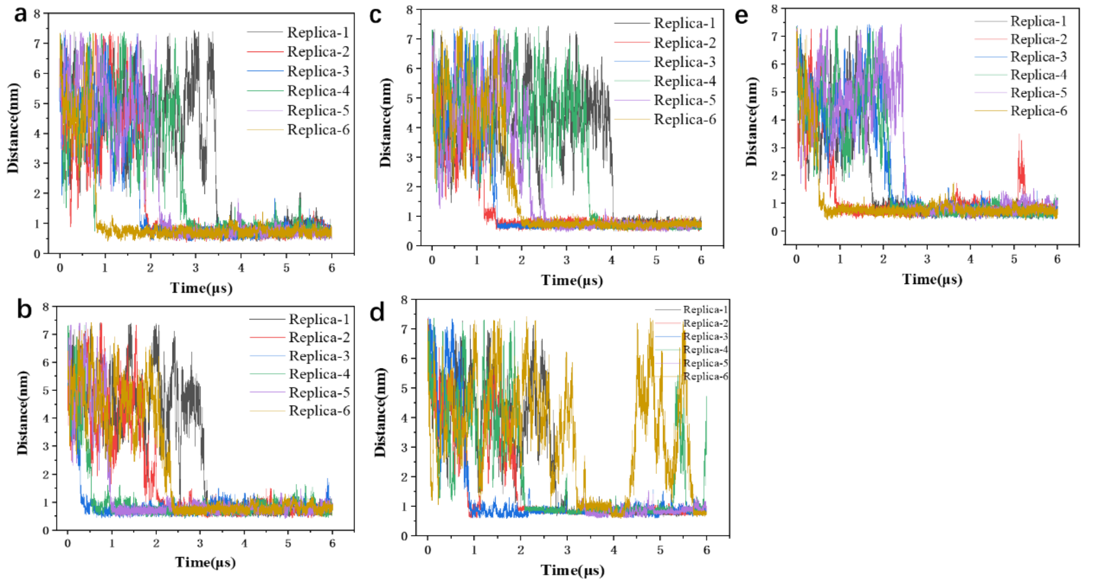

FIGURE S5. (a) Distance evolvements between the TM domains of Pal-286 in 2.2 version and (b) Pal-295 in 2.2 version, respectively. (c) Evolution of the distance between the TM domains of the C286AC295A mutation in 2.2 version and (d) in 3.0 version, respectively. (e) Evolution of the distance between the TM domains of the Pal-dual, respectively.

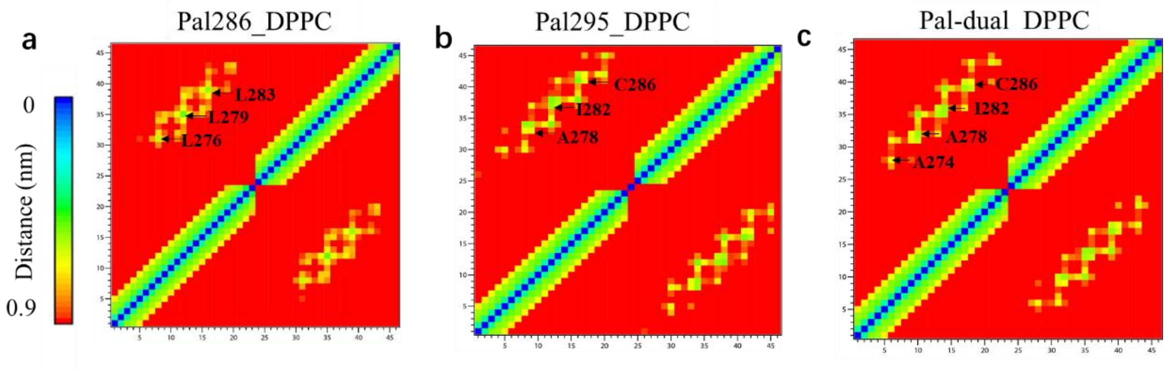

FIGURE S6. (a)-(c) The residues contacted matrixes of the predominant dimeric structures of Pal286, Pal295 and Pal-dual formed in the DPPC membranes.

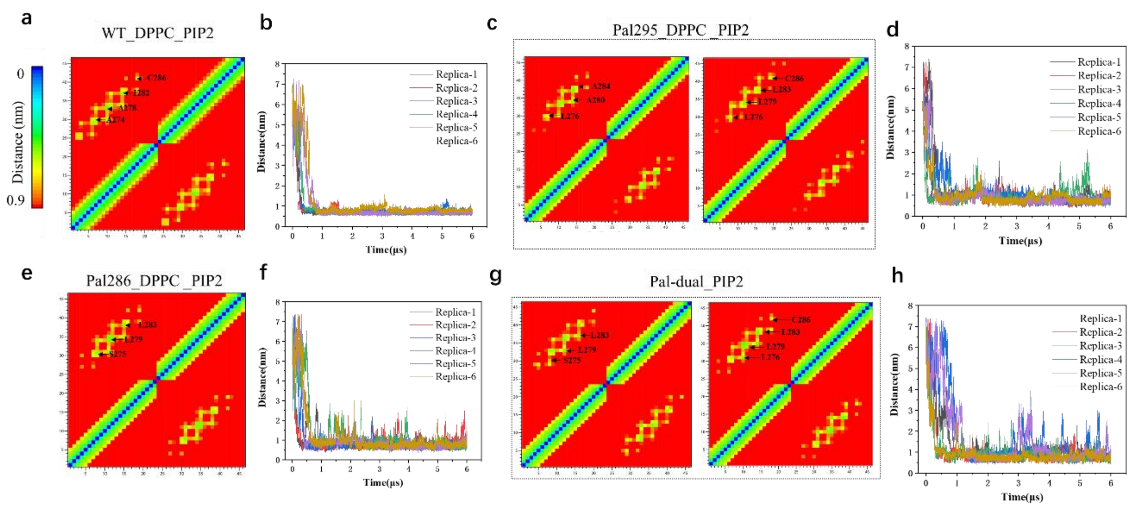

FIGURE S7. (a) The contact matrix distribution of WT predominant dimer residues in the PIP2-contained membrane. (b)

Distance evolvements between the TM domains of WT, respectively. (c) The contact matrix distribution of Pal295 predominant dimer residues in the PIP2-contained membrane. (d) Distance evolvements between the TM domains of Pal295, respectively. (e) The contact matrix distribution of Pal286 predominant dimer residues in the PIP2-contained. (f) Distance evolvements between the TM domains of Pal286, respectively. Membrane. (g) The contact matrix distribution of Pal-295 and Pal-dual predominant dimer residues in the PIP2-contained membrane. (h) Distance evolvements between the TM domains of Pal-295 and Pal-dual, respectively.

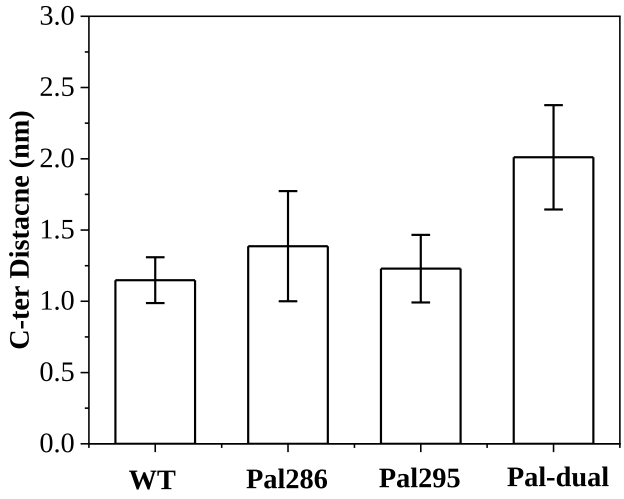

FIGURE S8. C-terminal distance of CD44-WT and the palmitoylated variants. The backbones of N290 were used for calculation.

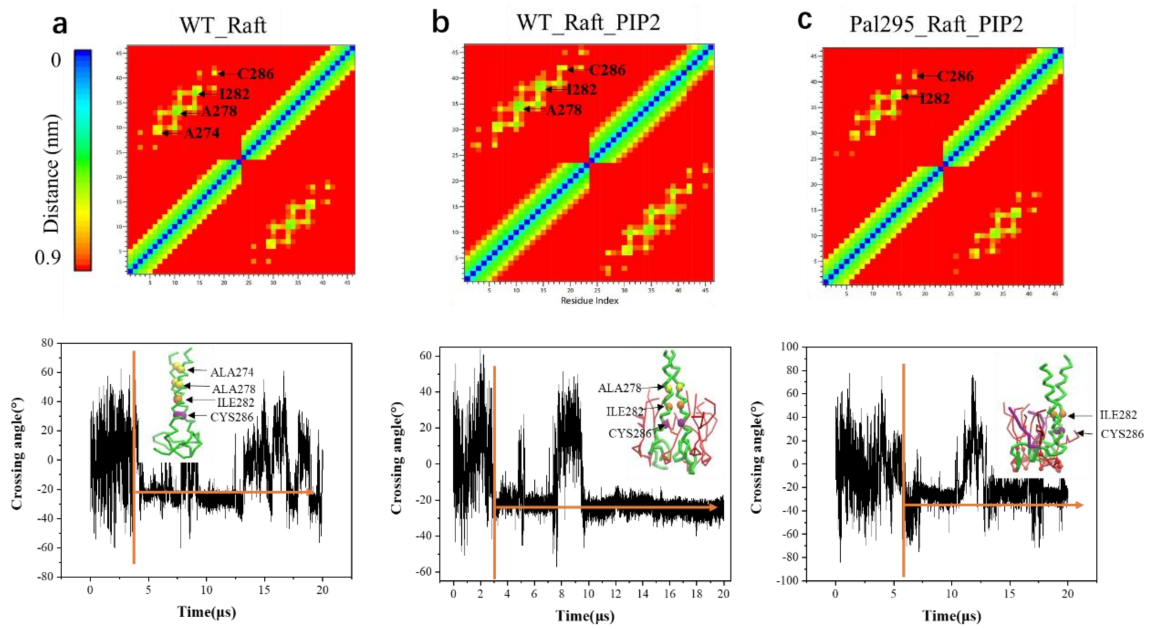

FIGURE S9. The upper panels: the residues contacted matrixes of predominant CD44 (WT and Pal-295) dimers formed in the lipid rafts, either in addition of PIP2 lipids. The lower panels represent the respective dimer structures. The color methods are consistence with the figures presented in the maintext.

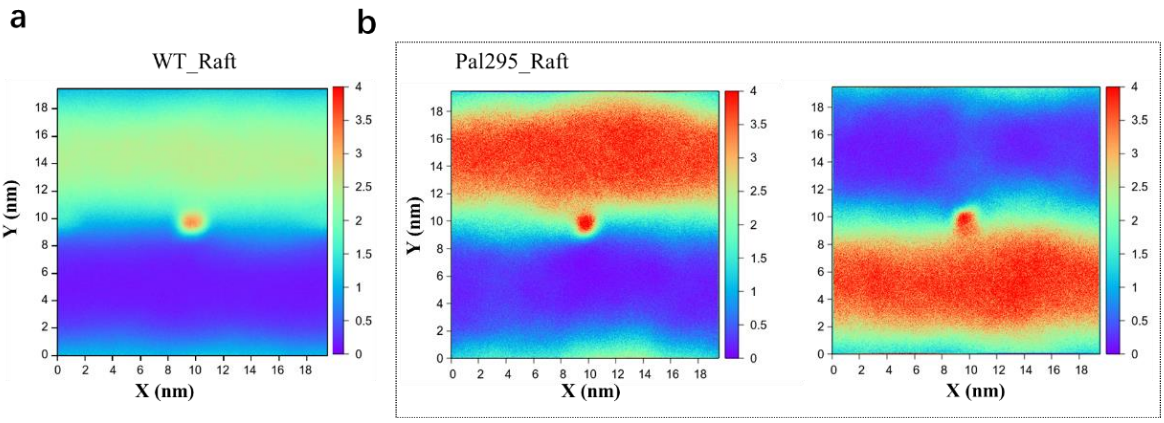

FIGURE S10. The figure shows the 2D density maps of the positions of the CD44-WT and Pal-295 monomers relative to the lipid rafts. Only the density of DPPC is taken into consideration to determine the position of the raft domain.

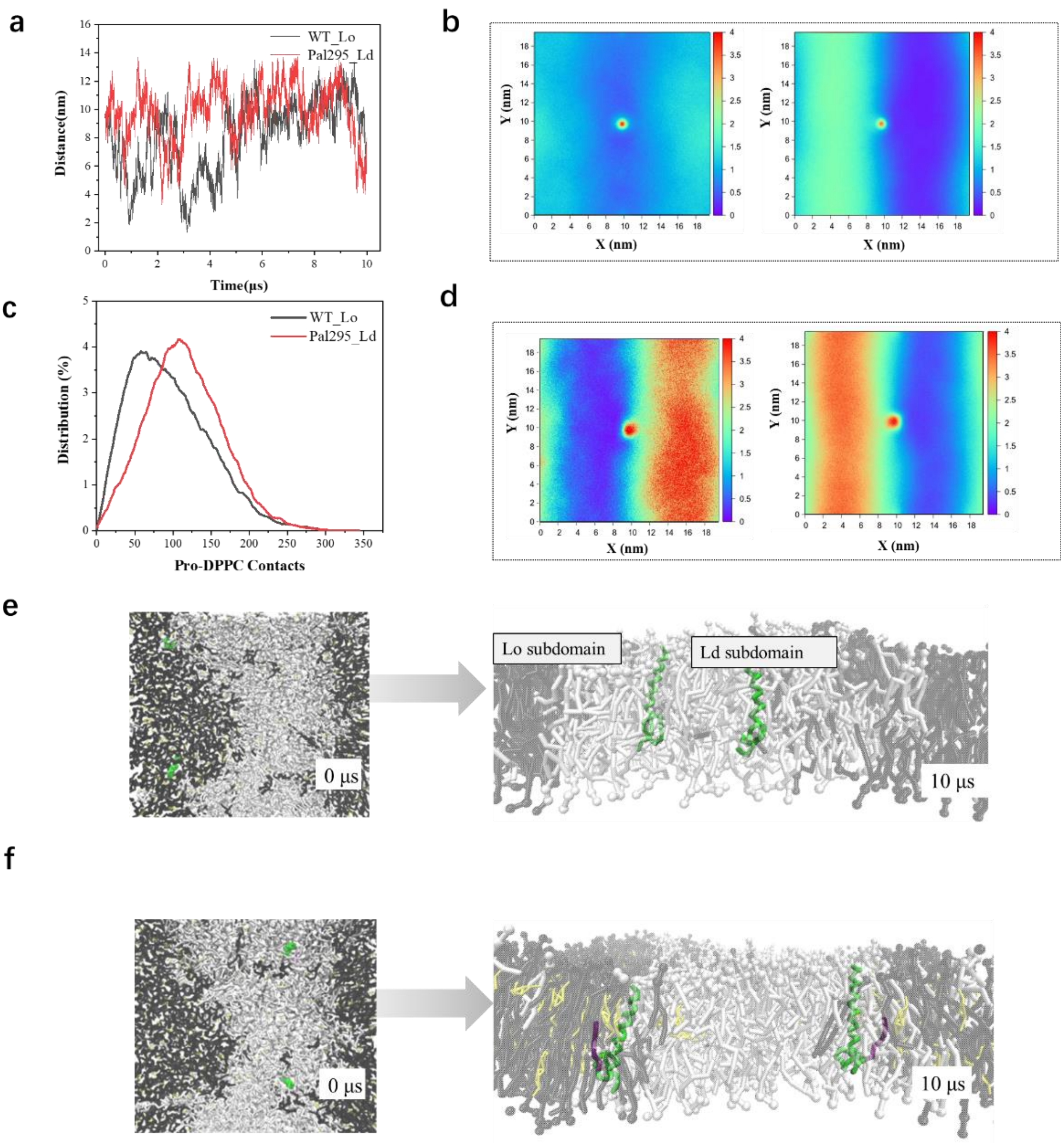

FIGURE S11. (a) Distance evolvements between the TM domains of WT_Lo and Pal-295_Ld, respectively. (b) Two-dimensional density maps of WT_Lo and (d) Pal-295_Ld relative to the lipid rafts. Only DPPC lipids were taken into consideration to position the raft domain. The heatmaps were optimized by the software package Gnuplot-5.4.0 (http://www.gnuplot.info/). (c) DPPC-Protein contact distributions of WT_Lo and Pal-295_Ld, respectively. (e) Localization

and dimeric orientation of WT_Lo and (f) Pal-295_Ld in the binary-phase membrane.

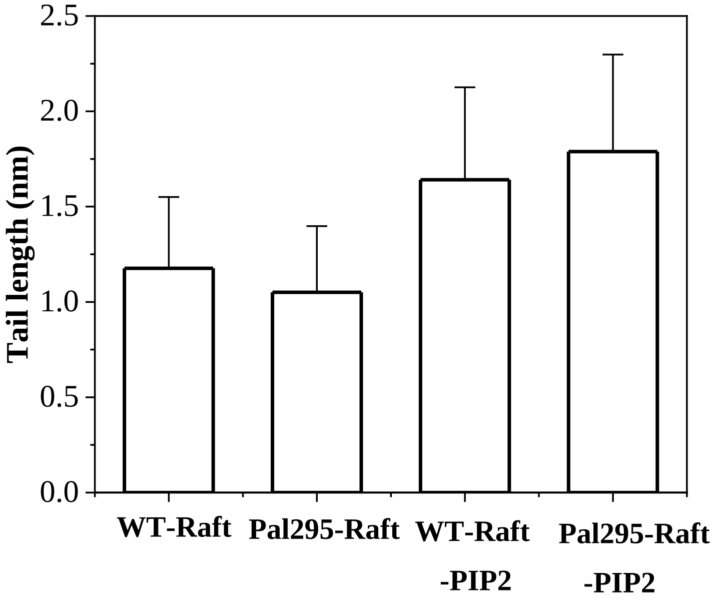

FIGURE S12. A comparison of cytoplasmic tail length of the CD44 and CD44-Pal295 in different membrane environments. The tail length is defined as this method: the distance between backbones of Arg-292 and Val-310 over simulation time is firstly calculated. All the distance points were then averaged and the standard deviations (caps here) were produced.

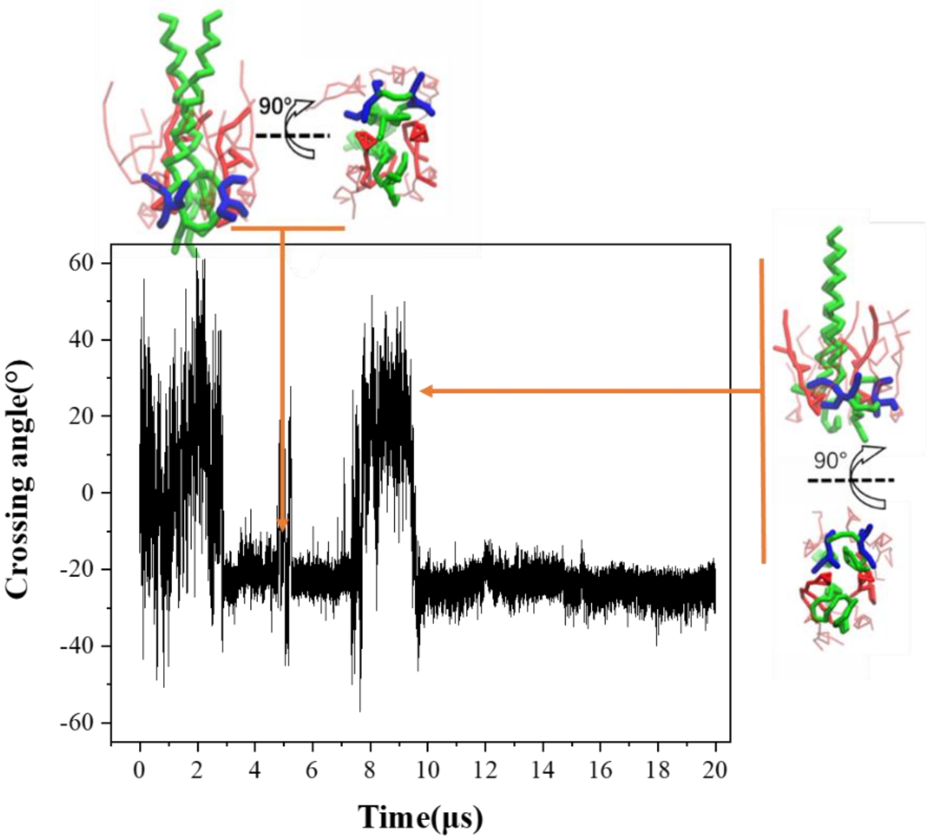

FIGURE S13. Crossing angle of CD44-WT in the PIP2-included phase-segregated membrane over the simulation time within 3-10 μs. Representative snapshots of the -20° RH conformation and parallel LH conformation are inserted in the panel. Two PIP2 molecules between the two peptides are bolded in red.

TABLE S1. The upper leaflet is composed of 100% DPPC lipids, while the lower leaflet contains either 100% DPPC lipids or 95% DPPC / 5% PIP2. The model of PIP2 was constructed with the *sn-1* chain fully saturated, and the *sn-2* chain poly-unsaturated, modeling C16:0-C18:4 PIP2 (4, 5). Detailed parameters of PIP2 can be seen in

our previous work. For the lipid raft formed membrane model is composed of 40% DPPC / 40% DIPC / 20% CHOL, which has been proved adaptive in forming phase-segregated bilayer. To study the effect of PIP2, only the lower leaf is replaced by 39% DPPC / 39% DIPC / 20% CHOL / 2% PIP2. CD44 is inserted vertically into the membrane, and ions (Na^+ or Cl^-) are added to neutral the systems.

An overview of the simulation system series presented in the study.

Protein series	Upper leaflet		Lower leaflet		W	Ions	Times	Box Size
	Lipids	Number	Lipids	Number			(μs)	
**CD44-TM**	100%DPPC	169	100%DPPC	169	4406	48(Na$^+$) 48(CL$^-$)	6	10×10×10 nm^3
**CD44-WT2.2 CD44-WT3.0**	100%DPPC	169	100%DPPC	169	4406	48(Na$^+$) 48(CL$^-$)	6	10×10×10 nm^3
**Pal-286**								
**Pal-295**								
**Pal-dual**								
**Mut2.2 Mut3.0**								
**CD44-WT**	100%DPPC	169	95%DPPC/ 5%PIP2	160/8	4709	52(Na$^+$) 52 (CL$^-$)	6	10×10×10 nm^3
**Pal-286**								
**Pal-295**								
**Pal-dual**								
**CD44-WT**	40%DPPC/40% DIPC/20%CHOL	270/270/ 135	40%DPPC/40% DIPC/20%CHOL	270/270/ 135	17701	12 (CL$^-$)	20	20×20×10 nm^3
**Pal-295**								
**CD44-WT**	40%DPPC/40% DIPC/20%CHOL	270/270/ 135	39%DPPC/ 39%DIPC/20.0% CHOL/2%PIP2	263/263/ 135/13	27893	40(Na$^+$)	20	20×20×10 nm^3
**Pal-295**								
